# Variability of Gene Expression After Polyhaploidization in Wheat (*Triticum aestivum* L.)

**DOI:** 10.1534/g3.111.000091

**Published:** 2011-06-01

**Authors:** Jiali Wang, Dongcheng Liu, Xiaoli Guo, Wenlong Yang, XiuJie Wang, Kehui Zhan, Aimin Zhang

**Affiliations:** * State Key Laboratory of Plant Cell and Chromosome Engineering, Institute of Genetics and Developmental Biology, Chinese Academy of Sciences, Beijing 100101, China; † College of life Science, China Agricultural University, Beijing 100193, China; ‡ State Key Laboratory of Plant Genomics, Institute of Genetics and Developmental Biology, Chinese Academy of Sciences, Beijing 100101, China; § Henan Agricultural University, Henan 450002, China

## Abstract

Interspecific hybridization has a much greater effect than chromosome doubling on gene expression; however, the associations between homeologous gene expression changes and polyhaploidization had rarely been addressed. In this study, cDNA–single strand conformation polymorphism analysis was applied to measure the expression of 30 homeologous transcripts in naturally occurring haploid (ABD, 2*n* = 21) and its polyploid maternal parent Yumai 21A (AABBDD, 2*n* = 42) in wheat. Only one gene (TC251989) showed preferentially silenced homoeoalleles in haploids. Further analyses of 24 single-copy genes known to be silenced in the root and/or leaf also found no evidence of homeologous silencing in 1-month-old haploids and two ESTs (BF484100 and BF473379) exhibit different expression patterns between 4-month-old haploids and hexaploids. Global analysis of the gene expression patterns using the Affymetrix GeneChip showed that of the 55,052 genes probed, only about 0.11% in the shoots and 0.25% in the roots were activated by polyhaploidization. The results demonstrate that activation and silencing of homoeoalleles were not widespread in haploid seedlings.

Polyploidy is a recurring process in the evolution of flowering plants that has had a considerable impact on plant species diversity (reviewed in Wendel and Doyle 2005). Estimates for the incidence of polyploidy in angiosperms vary from 30 to 80%, and 2–4% of speciation events can be attributed to genome duplications (Otto and Whitton 2000). According to the genome difference, polyploid can be classified as autopolyploidy (the doubling of a single genome) and allopolyploidy (the merger of two fully differentiated genomes) (Tate *et al.* 2004). Most of our important crop plants have evolved as a result of one or more wide hybridization events, each followed by a chromosome doubling step to restore fertility––the end product of these processes being a stable allopolyploid (Bottley *et al.* 2006).

In recent years, the consequences of polyploidy for the evolution of genes and genomes and for gene expression have been investigated extensively in plants (Song *et al.* 1995; Levy and Feldman 2004; Pires *et al.* 2004; Pontes *et al.* 2004; Udall *et al.* 2006; Wendel *et al.* 1995; Small *et al.* 1999). One direct and observable consequence of polyploidy is that homeologous genes are expressed at different levels and respond differently to allopolyploidy in various organs of the plants (reviewed in Adams 2007). Thus, a particular homoeoallele may be silenced in leaf tissue but expressed in the root, while another gene may have the opposite pattern of expression. Homeologous gene silencing in newly synthesized polyploids occurs at a frequency of approximately 5% in cotton and wheat (Adams *et al.* 2004; Kashkush *et al.* 2002) and at 0.5% in synthetic *Arabidopsis* alloploids (Comai *et al.* 2000). In contrast, in established polyploids, such as cotton, the proportion of genes with only partial homoeoalleles expressed is as high as 25% (Adams *et al.* 2003), while in hexaploid bread wheat, silencing occurs at a frequency of approximately 29% for the unigene loci, and typically only one of the three homoeoalleles present is silenced (Bottley *et al.* 2006). Homeologous gene expression patterns can vary by generation in neopolyploids (Wang *et al.* 2004), suggesting a sorting out process of expression regulation immediately after allopolyploidy that lasts for a few generations. The reactivation of silenced homoeoalleles, which has been observed in synthetic alloploids (Kashkush *et al.* 2002), in distinct plant organs of cotton (Adams *et al.* 2003), and among derived aneuploids of bread wheat (Bottley *et al.* 2006), indicates that homoeoalleles silencing is mainly achieved by epigenetic rather than genetic means.

Most studies on polyploidization have been focused on establishing the frequencies or patterns of homeologous gene expression in the context of polyploidization (Tate *et al.* 2004; Wendel and Doyle 2005; Adams 2007). Moreover, most of them employed the synthesized haploids and polyploids, while the natural polyploids were seldom used (Peng *et al.* 2008), except some recent polyploids such as *Spartina* (Baumel *et al.* 2001), *Tragopogon* (Cook and Soltis 1999) and *Senecio* (Abbott and Lowe 2004; Hegarty *et al.* 2006). As we know, both inducing treatment and tissue culture conditions have the potential to modify the DNA structure, influence gene expression, and eventually interference the accuracy of study on ploidy effects. For example, tissue culture can activate retrotransposon Tos17, and change the methylation status of its flank regions (Han *et al.* 2004), DNA sequence and gene expression (Cheng *et al.* 2006). Furthermore, to date the associations between homeologous gene expression changes and polyhaploidization had rarely been addressed, which might result in the inaccuracy for understanding ploidy effect in gene expression.

Yumai 21 is a male sterile line with the *Aegilops kotschyi* cytoplasm in wheat. *Ae. kotschyi* cytoplasm naturally induces the haploids form, which is free of inducing treatment or tissue culture, even in combinations between male-sterile wheat and a restoring line, the frequency of haploids is as high as 80% (Kobayashi 1980). Cytological observations and epigenetic studies provide evidence that the haploid originates from the female parent (Sun *et al.* 1994). Thus, this provides excellent material for elucidating the influence of polyhaploidization on homeologous gene expression patterns.

Homeologous sequences are, by definition, highly similar but nonidentical, and resolving individual homoeoallelic transcripts from one another has been technically difficult (Bottley *et al.* 2006). Pervious researchers have shown that the single-strand conformation polymorphism (SSCP) technique is capable of distinguishing homeologous wheat gDNA sequences (Forsström *et al.* 2003; Bottley *et al.* 2006). In the present study, we investigated the patterns of homeologous gene expression between the haploid and hexaploid wheats using the SSCP technique. Furthermore, the Affymetrix GeneChip was also exploited to survey the silencing and activation of genes after polyhaploidization. Our objective is to establish the extent to which patterns of homeologous silencing vary between haploids and its corresponding hexaploids.

## Materials and Methods

### Plant materials, RNA extraction, and cDNA synthesis

The haploid wheat (2*n* = 3*x* = 21 ABD, hereafter referred to as Yumai 21H) was obtained from a cross between the male-sterile wheat line Yumai 21 (2*n* = 6*x* = 42 AABBDD, hereafter referred to as Yumai 21A) with the *Aegilops kotschyi* Boiss cytoplasm and the maintainer line Yumai 21 (2*n* = 6*x* = 42 AABBDD, hereafter referred to as Yumai 21B) developed by Dr. Kehui Zhan (Henan Agricultural University, China). Seedlings were germinated on wet filter paper at 25°C, thereafter vernalized in the dark at 5°C for 18 d, and transplanted to soil (pots of Ø 17 cm) after vernalization. Plants were grown in a greenhouse at 24/18°C (day/night) with a 16-h photoperiod for 1 or 4 months. *T. turgidum* (AABB) and *Ae. tauschii* (DD) were used as controls of homeoallelic origin in the cDNA–SSCP experiments.

Shoot and/or root tissues from 1- or 4-month-old plants were harvested into liquid nitrogen prior to the extraction of total RNA and genomic DNA (gDNA). Total RNA were extracted using the TRIZOL reagent (Sigma Chemical Co.) according to the manufacturer's instructions, and crude RNA preparations were treated with DNase (Invitrogen), following a phenol/chloroform extraction (Sambrook and Russell 2001). cDNA was synthesized with Superscript II (Invitrogen), using oligo (dT) as the polyA primer and following the manufacturer's protocol. To detect the contaminating gDNA, newly synthesized cDNA was amplified with intron-spanning PCR primers (ActinF 5′-CTGCTTTGAGATCCACAT-3′, ActinR 5′-GTCACCACTTTCAACTCC-3′), which could distinguish the contaminating gDNA from the RNA-derived DNA. The entire experiment was performed with three independent biological replicates, and each biological sample was generated by pooling three individual plants together.

### Flow cytometric analysis

To determine the ploidy and confirm the haploid form, flow cytometry for nuclear content was conducted. Leaves or petals of each plant were subjected to flow cytometry following the method by Pfosser *et al.* (1995) and analyzed with a BD FACSCalibur (BD Biosciences).

### EST selection, PCR amplification, and SSCP analysis

To investigate the regulation of homeologous gene expression between the haploid and hexaploid wheat, two sets of expressed sequence tags were exploited. First, 30 low-copy ESTs were selected from the previous study of Pumphrey *et al.* (2009) on the basis that they had been verified by Southern blot hybridization to have the same number of deletion bins (*e.g.* having three bands, with one band from each homeologous locus in a group of chromosomes). An additional 24 ESTs that mapped exclusively to a set of homeologous genes located on one of the wheat chromosome groups 1, 2, 3, or 7 were arbitrarily selected from those examined by Bottley *et al.* (2006) on the basis that they had clearly defined SSCP profiles and exhibited silencing in either the Florida or Chinese Spring cultivars. RT-PCR was carried out in a volume of 10 μl, which comprised 1 μl cDNA template (1:20 dilution of the cDNA), 5 μl Hotstar Master Mix (Qiagen), 0.25 μl of each primer (10 mm concentration), and 3.5 μl distilled water. The PCR program consisted of a predenaturation step of 15 min at 95°C, followed by 35 cycles of 95°C for 30 sec, 59°C for 30 sec, and 72°C for 60 sec, and a final extension step of 72°C for 10 min. Amplicons were electrophoretically separated by SSCP and visualized by silver staining (Bottley *et al.* 2006).

### Pattern analysis

The SSCP profiles generated from the gDNA were compared with those derived from the equivalent cDNA template. If all the homeologous copies identified for the gDNA template were also present in the cDNA profile, full expression was assumed for the homeologous sequences. For cases in which, despite the known presence of a gDNA locus, no matching cDNA was detected, the relevant locus was scored as “silenced.” For all the homoeoloci, duplicate assays were carried out to confirm the data.

### Sampling and RNA extraction for Affymetrix GeneChip hybridization

One-month-old plants were used to evaluate the differences in gene expression patterns between haploids and its parent, Yumai 21A, with Affymetrix GeneChip hybridization. Shoot and /or root tissues of each plant were sampled and frozen immediately, and sent to Genetimes Technology Inc. (Shanghai, China) for cDNA synthesis. Labeled target used in hybridizations was generated from 0.5 μg of total RNA using the One-Cycle Eukaryotic Target Labeling Assay. Hybridizations, washing, staining and scanning were performed as specified in the manufacturer’s protocol (http://www.affymetrix.com). The hybridization was performed with three independent biological replicates, and each biological sample was a pool of three plants.

### Affymetrix GeneChip data analysis

Fluorescence intensity data were recorded using GenePix Pro v6.0 (Molecular Devices) (supporting information, Table S1). Lower-quality and weakly hybridizing features were stringently flagged for exclusion (by requiring a circularity ratio ≥0.8, signal intensity >75% of background plus one standard deviation, and the sum of median intensity from each channel >900) to initially reduce printing and hybridization errors. Data normalization was performed according to Bolstad's quantile procedure for oligonucleotide arrays (Bolstad *et al.* 2003). Therefore, control and empty spots were filtered out before normalization. Statistical analysis was performed with GeneSpring 7.3 (Agilent Technologies) using methods adapted from Casu *et al.* (2007). Present (P) and absent (A) calls for Affymetrix probe sets are generated based on statistical analysis of hybridization signal of oligonucleotides from one probe set where present (*P* < 0.04) and absent (*P* > 0.06).

## Results

### Haploids screening

**Figure 1:**
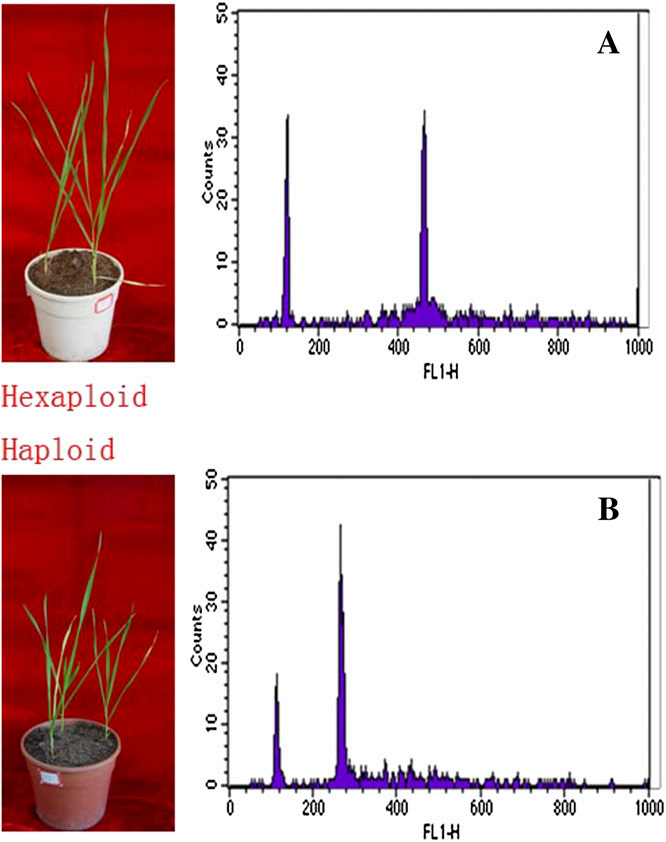
Flow cytometric analysis of different ploidy levels in wheat. (A) Hexaploid. (B) Haploids.

Although the number of chromosomes in the haploid wheat was half of that in the hexaploids, they exhibited similar morphologies in the seedling stage (Figure 1); therefore, it is difficult to distinguish haploids from hexaploids in wheat. Therefore, we applied flow cytometry to screen haploids. Examination using the leaves of confirmed haploid and hexaploid wheat plants showed that the fluorescence peak of hexaploid nuclei located at channel position 400, whereas that of haploid nuclei located at channel position 200 (Figure 1A), consistent with the expected DNA relationship of the two types of plants. Among the 282 individuals analyzed by flow cytometry, 27 samples had nuclei with a fluorescence peak at channel position 200 (not accounting for twin seedlings), which were considered as haploids, and were confirmed by our observations of cytology and morphology (Figure S1).

#### Detection of differential expression of genome homoeoalleles by cDNA–SSCP:

**Figure 2:**
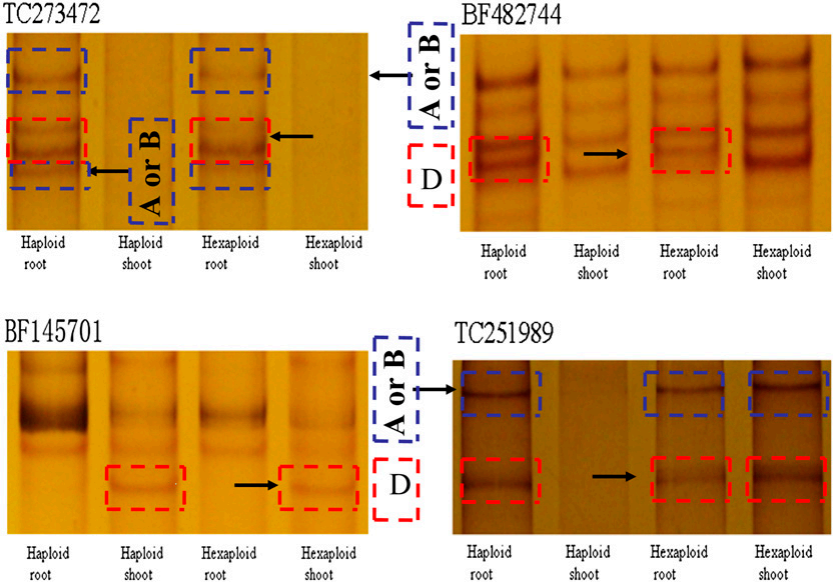
Organ-specific transcripts silencing of four ESTs identified from the cDNA–SSCP analysis of 30 expressed sequence tags in seedlings. Equal amounts of second-strand cDNA from a hexaploid (AABBDD) and a naturally produced polyhaploids (ABD) were PCR-amplified with conserved primers for each locus, and electrophoresed on MDE polyacrylamide gels. The arrows indicate the bands from different genome.

The expression patterns of individual genome homoeoalleles were assessed by cDNA–SSCP with seedlings of Yumai 21A and Yumai 21H. A set of 30 well-characterized transcripts (Pumphrey *et al.* 2009) was first used for comparison and validation. These transcripts had clearly defined SSCP profiles. Unexpectedly, of the thirty low-copy ESTs, 29 have similar electrophoretograms between the haploid and the hexaploid forms in both the root and shoot tissues, revealing that neither activation nor silencing of the genome homoeoalleles was detected following polyhaploidization (Figure 2). Only one EST (TC251989) showed altered expression pattern between the haploids and the hexaploids. The A or B and D genome homoeoalleles of TC251989^p^ (^p^ indicated primers that were selected from Pumphrey *et al.* 2009. ESTs without this tag were selected from Bottley *et al.* 2006) were expressed in both the shoot and root tissues of the hexaploids, and expressed in root but not in shoot of haploids (Figure 2). These results revealed that all the homoeoalleles of TC251989^p^ were silenced in the shoot tissues after polyhaploidization.

Organ-specific expression was detected in the hexaploids and haploids. Three of the 30 arbitrarily selected sets of homeologous transcripts (10%) displayed organ-specific expression patterns in the hexaploid seedlings. As shown in Figure 2, the D genome homoeoallele of BF482744^p^ was not expressed in the shoot tissue of the hexaploids, whereas the A, B and D genome homoeoallele of TC273472^p^ were absent in shoot tissue. To the contrary, the D genome homoeoallele of BF145701^p^ was lacking in the root tissue of the hexaploid seedlings. A similar expression pattern was observed for haploids (Figure 2).

**Figure 3:**
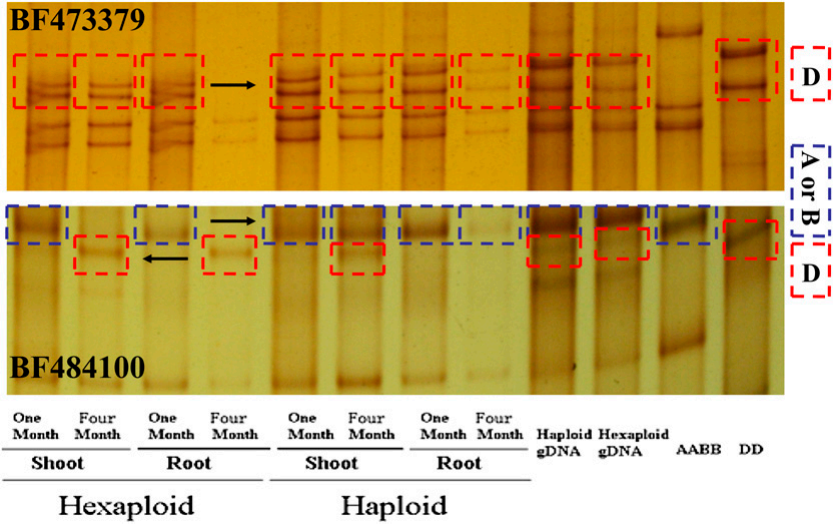
Images of the specifically expressed EST transcripts, as identified by cDNA–SSCP analysis of 24 arbitrary selected expressed sequence tags. Equal amounts of second-strand cDNA from a hexaploid (AABBDD) and a naturally produced polyhaploids (ABD) were PCR-amplified with conserved primers for each locus, and electrophoresed on MDE polyacrylamide gels. The arrows indicate the bands from different genome.

To increase the number of genes surveyed, we assayed an additional 24 ESTs of which at least one homoeoallele is known to be silenced in the variety of Chinese Spring (Bottley *et al.* 2006). Direct comparisons were possible between gDNA profiles and their corresponding cDNA-derived profiles because the amplicons represent intron-free sequences. There were no significant changes in the numbers of genome homoeoalleles between the 1-month-old haploid and hexaploid seedlings. For example, the B genome homoeoallele of BF499478 (only one copy was identified in Chinese Spring) was detected in all tissues of hexaploid seedlings. Similar expression pattern of this homoeoallele was also present in all tissues of haploid seedlings (Figure S2). Furthermore, to understand the homeologous gene expression in different developmental stage, the expression patterns of 24 ESTs in 4-month-old haploids and hexaploids were investigated. Comparison of the cDNA and gDNA profiles revealed that the D genome homoeoallele of BF473379 was not in the root tissue of hexaploids but present in haploids, and was therefore activated in the shoot sample (Figure 3). The A or B genome homoeoallele of BF484100, which was apparently not expressed in either the shoot or root tissues of hexaploids, became active in both tissues of haploids. An unexpected observation was that the D genome homoeoallele of BF484100 was present in shoots but not detected roots of haploids. These varied expression patterns suggest that there was a bias toward selective and transcriptional inactivation of some homoeoalleles.

**Figure 4:**
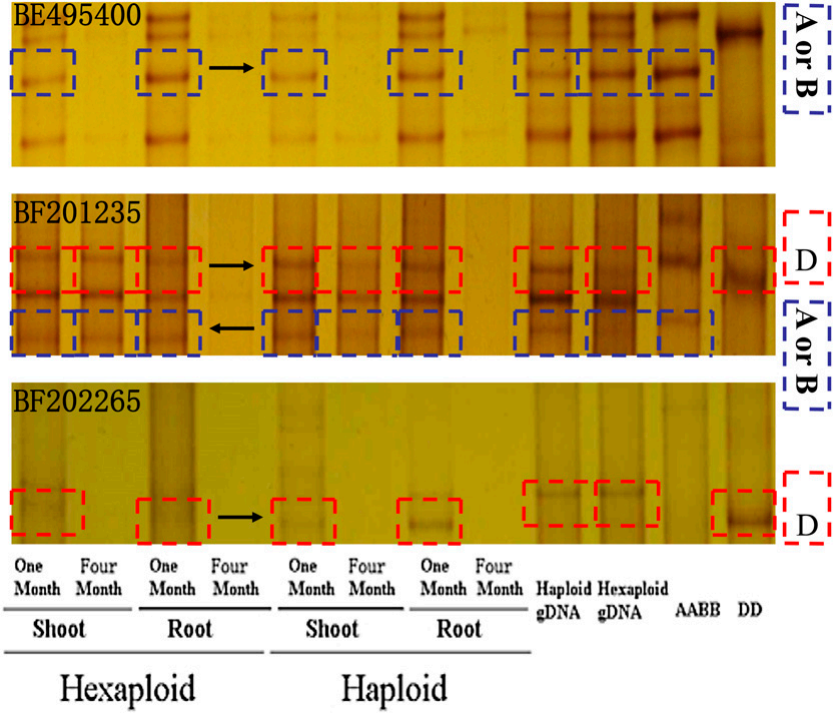
cDNA–SSCP analysis of differentially expressed transcript with respect to a developmental process. Equal amounts of second-strand cDNA from a hexaploid (AABBDD) and a naturally produced polyhaploids (ABD) were PCR-amplified with conserved primers for each locus, and electrophoresed on MDE polyacrylamide gels. The arrows indicate the bands from different genome.

Developmentally regulated gene silencing occurs both in haploids and hexaploids. Of the 24 arbitrarily selected sets of ESTs, four in haploids and five in hexaploids showed developmentally regulated silencing (Figures 3 4). For instance, the A or B genome homoeoallele of BE495400 and D genome homoeoallele of BF202265 were expressed in 1-month-old tissues of the hexaploids, but not expressed in 4-month-old samples. Similar expression patterns of these two ESTs were also observed in haploids (Figure 4). At the same time, full expression of all homoeoalleles of BF201235 were observed in both the 1- and 4-month-old shoot and 1-month-old root but not in the 4-month-old root of hexaploids and haploids.

#### Estimation of gene expression by microarray analysis:

To further understand the regulation of gene expression in polyhaploidization, we performed a global analysis of gene expression using the Affymetrix GeneChip. The microarray hybridization results revealed that a large number of loci had increased or decreased expressions after Yumai 21A polyhaploidization. Because the objective of this study was to exploit gene silencing and activation in polyhaploidization, we mainly focused on genes specifically expressed in either the haploid or hexaploid plants. The locus only expressed in haploids is defined as activated, reversely, as silenced. Among the 55,052 detected transcripts, 64 loci were activated and 227 loci were silenced in the shoot tissue of haploids, and the corresponding numbers were 138 loci and 28 loci in the roots (Table 1). The low numbers of loci being activated or silenced in haploids as compared to hexaploids confirmed our hypothesis that activation or silencing of homeologous transcripts are not the major strategy used for gene expression regulations after polyhaploidization (Table 2).

**Table 1:** Numbers of sensitive loci changed in expression patterns after polyhaploidization

Alteration Pattern	Up-regulation		Down-regulation		Activated		Silenced	
Material	Shoot	Root	Shoot	Root	Shoot	Root	Shoot	Root
Number	312	334	448	69	64	138	227	28
Ratio in chip (%)	0.57	0.61	0.81	0.12	0.11	0.25	0.41	0.04

**Table 2:** Single-copy EST loci silenced in 4-month-old haploids and hexaploids

GenBank ID	Function/Putative Function	Homoeoallele Silenced in CS	Tissue	Genomes Silenced			
				Haploids		Hexaploids	
				Shoot	Root	Shoot	Root
BE495400	Unknown	B	ROOT	A/B	A/B	A/B	A/B
BF201235	*Rubisco* subunit binding protein α subunit	D	LEAF		A/BD		A/BD
BF202265	Unknown	A	LEAF	D	D	D	D
BF473379	Unknown	D	LEAF				D
BF484100	Unknown	D	LEAF		D	A/B	A/B

## Discussion

Haploidy species rarely exist in nature. Almost all the extant haploids are created by the techniques such as another culture or parthenogenesis (Peng *et al.* 2008). However, the origination of haploid (Yumai 21H) was a mild process, which was free of inducing treatment or tissue culture and thus avoids artificial introduction of mutations. As a result, the ploidy series, which spontaneously originated under natural conditions, can partially imitate the natural ones and be used to precisely estimate ploidy effects in gene expression of crop polyploids.

Four of the 30 arbitrarily selected sets of homeologous transcripts (∼13%) showed altered expression patterns in a synthetic *T*. *aestivum* line and the *T*. *turgidum* and *Ae. tauschii* parents (Pumphrey *et al.* 2009). Suppression of *Ae. tauschii* D genome homoeoallele in synthetic *T*. *aestivum* line was observed for two differentially expressed transcripts, TC270558^p^ and TC273936^p^. However, in our study, these two transcripts did not display altered expression patterns in the haploids, as compared with the hexaploids. Of the 30 arbitrary selected sets of homeologous transcripts, only one transcript (TC251989^p^) displayed different expression patterns in the haploid and its corresponding hexaploid form. Amplification and electrophoresis of a further 24 ESTs, for which at least one homoeoallele was known to be silenced in the variety Chinese Spring (Bottley *et al.* 2006), showed that there were no significant changes in the numbers of genome homoeoalleles between the haploid and hexaploid seedlings. Homoeoalleles silenced in Chinese spring (either leaf or root) were not become reactive in polyhaploidization. For instance, the A genome homoeoallele of EST BE426364 was silent in leaf tissue of Chinese spring (CS hexaploid), but it was not restored in haploids. A comparison of the expression patterns of 24 ESTs in a panel of wheat varieties appears in Table S2. Furthermore, our electrophoresis patterns of the homoeoalleles revealed that the expression of at least one copy of five ESTs (BE495400, BF484100, BF473379, BF201235, and BF202265) varied qualitatively in either the root or shoot tissue of the 4-month-old hexaploid with respect to a developmental process (Figures 3 and 4). Unexpectedly, a similar expression patterns for the three ESTs were also observed in the haploids (BE495400, BF201235, and BF202265). The expression patterns of these ESTs were regulated with respect to a developmental process, however, were not altered by polyhaploidization. All these results showed that the silencing or reactivation of homoeoalleles after haploidisation during a specific phase of haploids development is not widespread.

To eliminate potential bias in the data collected using cDNA–SSCP, we performed a global analysis of the gene expression patterns of haploid and hexaploid seedlings. Of the 55,052 genes probed, only about 0.41% in the shoots and 0.04% in the roots were silenced by polyhaploidization. This contrast with the high proportion of 5% of genes in newly synthesized wheat hexaploids (Kashkush *et al.* 2002) and 7–8% of genes in established wheat hexaploids (He *et al.* 2003) are silenced. One reason for these contradictions is that allopolyploids are often derived from a 2-step process: hybridization between 2 species and then doubling of all the chromosomes by the union of unreduced gametes (or by other mechanisms) (reviewed in Adams 2007). However, the polyhaploidization process is like the reversed way of autopolyploidy. Thus it cannot be directly compared with the allopolyploidization process and its effect on gene expression. Several studies have indicated that the effect of chromosome doubling on gene expression is much more insignificant than that of interspecific hybridization (Hegarty *et al.* 2006; Wang *et al.* 2006). For example, comparative proteomics of synthetic *B. napus* and its homozygous diploid progenitors *B. rapa* and *B. oleracea* showed that very few proteins disappeared or appeared in the amphiploids (<1%), but a strikingly high number (25–38%) of polypeptides displayed quantitative nonadditive pattern (Albertin *et al.* 2006). Results in this study reveal that a set of chromosomal deletions are comparable with autopolyploidization in plants, which also has a minor effect on gene expression.

Our results also reveal that the expression profiles of D genome homoeoallele are most frequently altered in haploids and hexaploids (Table 2). Bottley *et al.* (2006) reported a modest bias toward silencing of the D genome copies among leaf transcripts, but not among root transcripts. He *et al.* (2003) found that the homoeoalleles of D genomes were silenced twice as frequently as those from A or B genomes in a newly synthesized wheat hexaploid line. In contrast, no pattern of preferential silencing was detected in the newly synthesized wheat hexaploid × hexaploid *Aegilops* hybrids (Kashkush *et al.* 2002). However, neither study provides evidence to support the hypothesis that there is a tendency toward selective silencing of homoeoallele from any one genome (Bottley and Koebner 2008; Bottley *et al.* 2008).

The production of haploid plants and homozygous double-haploids of crop species accelerates breeding programs, improves selection efficiency, and facilitates genetic analysis (Sharma *et al.* 2002). When working with plants that are normally polyploid, it is very useful to work at a low ploidy level through haploid induction (Carputo and Barone 2005). In plants, the transition from hexaploids to haploids involves modifications to the phenotype, although the haploid form carries the same genetic material as the female parent. The haploid plants are dwarfish in height, show weaker growth, and have smaller flowers than the diploid plants (Belicuas 2007). However, the results of our experiments suggest that activation or silencing genome homoeoalleles are not the major effect of polyhaploidization on gene expression. Our findings raise the important issue of what causes the reduction in growth potential of the haploid. Further studies of polyhaploidization are required to explore the effects of gene dosage on gene expression in haploid forms.

## Supplementary Material

Supporting Information
