# Supplementary material for: Variability of Gene Expression After Polyhaploidization in Wheat (Triticum aestivum L.)
Source: G3 (Bethesda). 2011 Jun 1;1(1):27–33. doi: 10.1534/g3.111.000091 (PMC3276123; doi:10.1534/g3.111.000091)
Supplement: Supporting Information [file supp_1_1_27__index.html]

Supporting Information 

# Variability of Gene Expression After Polyhaploidization in Wheat (*Triticum aestivum* L.)

## Supporting Information for Wang *et al.*, 2011

**Files in this Data Supplement:**

- Supporting Information - Figures S1 and S2 and Tables S1 and S2 (PDF, 460 KB)
- Figure S1 - Karyotype analysis in root tips of the haploid wheat (PDF, 228 KB)
- Figure S2 - Images of the expression patterns of two EST transcripts, as identified by cDNA-SSCP analysis (PDF, 156 KB)
- Table S2 - A comparison among genes shown to be expressed in leaf and/or root tissue of Chinese Spring (CS) (Bottley et al, 2006), the presence or absence of homoeologue silencing in the cultivar Florida (Bottley et al, 2008) and the expression of the same genes in callus tissue (PDF, 52 KB)
- Table S1 - Fluorescence intensity data (Compressed Excel file, .zip, 4.7 MB)
