# Supplementary material for: Variability of Gene Expression After Polyhaploidization in Wheat (Triticum aestivum L.)
Source: G3 (Bethesda). 2011 Jun 1;1(1):27–33. doi: 10.1534/g3.111.000091 (PMC3276123; doi:10.1534/g3.111.000091)
Supplement: Supporting Information [file supp_1.1.27_000091SI.pdf]

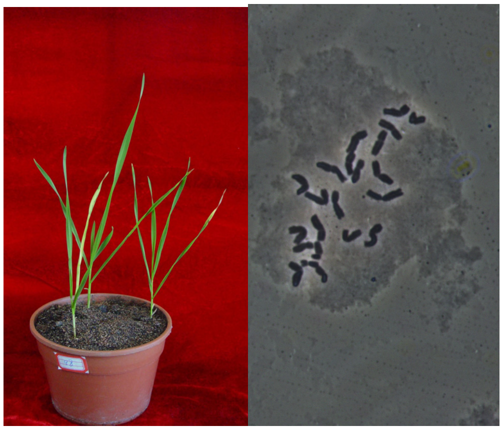

Figure S1 Karyotype analysis in root tips of the haploid wheat. Cytological preparations were carried out on root tips obtained from seeds germinated on sterile moist filter paper in Petri dishes at 25°C. Roots were pretreated with 0.05% colchicine solution for 2–3 h. and fixed in Carnoy for 24 h. and stored in 70% ethanol at 4°C.

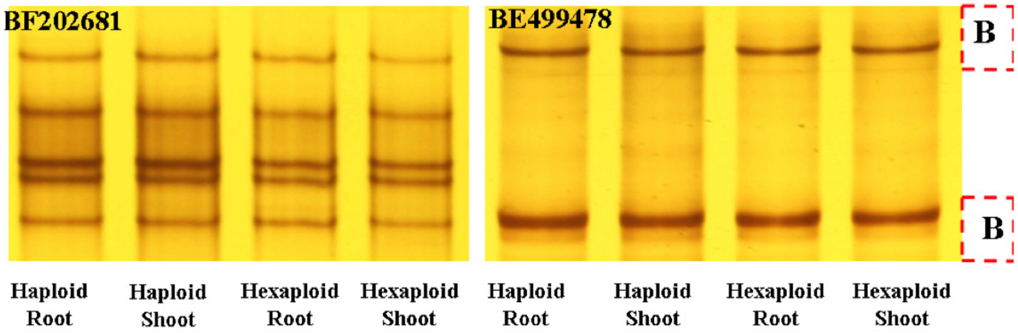

Figure S2 Images of the expression patterns of two EST transcripts, as identified by cDNA–SSCP analysis. Equal amounts of second-strand cDNA from a hexaploid (AABBDD) and a naturally produced polyhaploids (ABD) were PCR-amplified with conserved primers for each locus, and electrophoresed on MDE polyacrylamide gels. The arrows indicate the bands from different genome.

Table S1 Fluorescence intensity data

Table S1 is available for download as a compressed Excel file at http://www.g3journal.org/lookup/suppl/doi:10.1534/g3.111.000091/-/DC1/TableS1.zip.

Table S2 A comparison among genes shown to be expressed in leaf and/or root tissue of Chinese Spring (CS) (Bottley et al, 2006), the presence or absence of homoeologue silencing in the cultivar Florida (Bottley et al, 2008) and the expression of the same genes in callus tissue.

Genbank id	Expressed Callus	Homoeologue silenced in CS	Tissue	Homoeologues identifiable in CS	Homoeologue silenced in Florida	Putative function
BE399113	Y	D/D	LEAF/ROOT	B D	B	Unknown
BE444894	Y	D/B	LEAF/ROOT	A B D		saline responsive OSSRIII protein
BF482273	Y	D/B	LEAF/ROOT	B D		Unknown
BF201235	N	D	LEAF	A B D		Rubisco subunit binding-protein alpha subunit
BF473379	Y	D	LEAF	B D	D	Unknown
BF478825	Y	D	LEAF	A B D		Unknown
BF484100	N	D	LEAF	A B D	B	Unknown
BM138439	Y	D	ROOT	A B D		
BE443527	N	B/B	LEAF/ROOT	A B D	B	Unknown
BE404371	Y	B	ROOT	B D		NADH glutamate dehydrogenase
BE495400	N	B	ROOT	A B D	A	Unknown
BE499478	Y	B	ROOT	B +	B	FAT domain-containing protein/phosphatidylinositol 3- and 4-kinase family protein
BF202681	Y	B	ROOT	A B		Unknown
BE426364	Y	A/A	LEAF/ROOT	A D	A	glyceraldehyde-3-phosphate [illegible]
BE591763	Y	A	LEAF	A B D		Unknown
BF202265	Y	A	LEAF	A D		Unknown
BE500510	Y	-	-	-		
BE591372	Y	-	-	-		
BE638105	Y	-	-	-		
BM136908	Y	-	-	-		

The symbol '-' denotes that the homoeologous gene set is not afflicted by silencing. EST sequences blasted against NCBI Nucleotide collection
