# Supplementary material for: Variability of Gene Expression After Polyhaploidization in Wheat (Triticum aestivum L.)
Source: G3 (Bethesda). 2011 Jun 1;1(1):27–33. doi: 10.1534/g3.111.000091 (PMC3276123; doi:10.1534/g3.111.000091)
Supplement: Supporting Information [file supp_1.1.27_FigureS1.pdf]

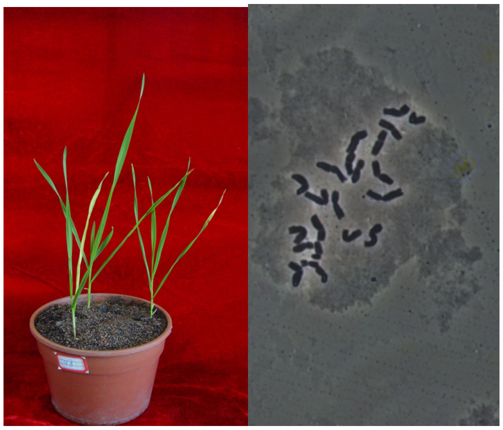

**Figure S1** Karyotype analysis in root tips of the haploid wheat. Cytological preparations were carried out on root tips obtained from seeds germinated on sterile moist filter paper in Petri dishes at 25°C. Roots were pretreated with 0.05% colchicine solution for 2–3 h. and fixed in Carnoy for 24 h. and stored in 70% ethanol at 4°C.
