# Supplementary material for: Variability of Gene Expression After Polyhaploidization in Wheat (Triticum aestivum L.)
Source: G3 (Bethesda). 2011 Jun 1;1(1):27–33. doi: 10.1534/g3.111.000091 (PMC3276123; doi:10.1534/g3.111.000091)
Supplement: Supporting Information [file supp_1.1.27_FigureS2.pdf]

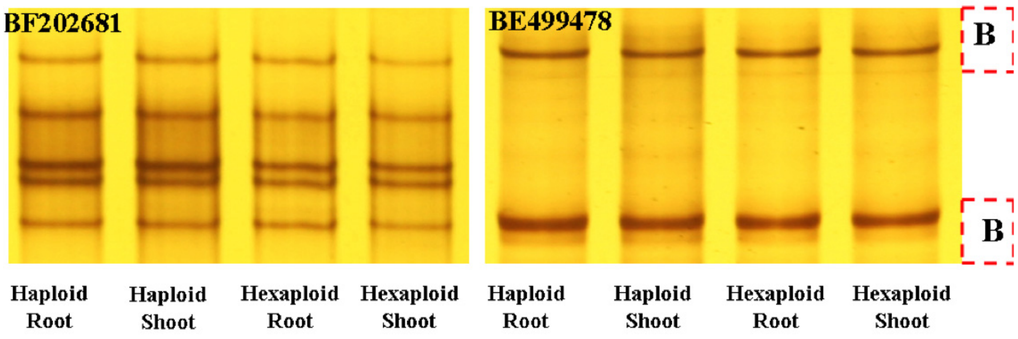

Figure S2 Images of the expression patterns of two EST transcripts, as identified by cDNA–SSCP analysis. Equal amounts of second-strand cDNA from a hexaploid (AABBDD) and a naturally produced polyhaploids (ABD) were PCR-amplified with conserved primers for each locus, and electrophoresed on MDE polyacrylamide gels. The arrows indicate the bands from different genome.
