# Supplementary material for: Variability of Gene Expression After Polyhaploidization in Wheat (Triticum aestivum L.)
Source: G3 (Bethesda). 2011 Jun 1;1(1):27–33. doi: 10.1534/g3.111.000091 (PMC3276123; doi:10.1534/g3.111.000091)
Supplement: Supporting Information [file supp_1.1.27_TableS2.pdf]

Table S2 A comparison among genes shown to be expressed in leaf and/or root tissue of Chinese Spring (CS) (Bottley et al, 2006), the presence or absence of homoeologue silencing in the cultivar Florida (Bottley et al, 2008) and the expression of the same genes in callus tissue.

Genbank id	Expressed Callus	Homoeologue silenced in CS	Tissue	Homoeologues identifiable in CS	Homoeologue silenced in Florida	Putative function
BE399113	Y	D/D	LEAF/ROOT	B D	B	Unknown
BE444894	Y	D/B	LEAF/ROOT	A B D		saline responsive OSSRIII protein
BF482273	Y	D/B	LEAF/ROOT	B D		Unknown
BF201235	N	D	LEAF	A B D		Rubisco subunit binding-protein alpha subunit
BF473379	Y	D	LEAF	B D	D	Unknown
BF478825	Y	D	LEAF	A B D		Unknown
BF484100	N	D	LEAF	A B D	B	Unknown
BM138439	Y	D	ROOT	A B D		
BE443527	N	B/B	LEAF/ROOT	A B D	B	Unknown
BE404371	Y	B	ROOT	B D		NADH glutamate dehydrogenase
BE495400	N	B	ROOT	A B D	A	Unknown
BE499478	Y	B	ROOT	B +	B	FAT domain-containing protein/phosphatidylinositol 3- and 4-kinase family protein
BF202681	Y	B	ROOT	A B		Unknown
BE426364	Y	A/A	LEAF/ROOT	A D	A	glyceraldehyde-3-phosphate [illegible]
BE591763	Y	A	LEAF	A B D		Unknown
BF202265	Y	A	LEAF	A D		Unknown
BE500510	Y	-	-	-		
BE591372	Y	-	-	-		
BE638105	Y	-	-	-		
BM136908	Y	-	-	-		

The symbol '-' denotes that the homoeologous gene set is not afflicted by silencing. EST sequences blasted against NCBI Nucleotide collection
